# Perception of quality of life in people experiencing or having experienced a *Clostridioides difficile* infection: a US population survey

**DOI:** 10.1186/s41687-020-0179-1

**Published:** 2020-02-19

**Authors:** Lise Lurienne, Pierre-Alain Bandinelli, Thibaut Galvain, Charles-Alexis Coursel, Caterina Oneto, Paul Feuerstadt

**Affiliations:** 1 Da Volterra, 172 rue de Charonne, 75011 Paris, France; 2 Concorde Medical Group, New York, USA; 3 Yale School of Medicine, New Haven, CT USA; 4 Gastroenterology Center of Connecticut, Hamden, CT USA

**Keywords:** *C. difficile* infection (CDI), Quality of life, Recurrence, Psychological consequences, Survey, Questionnaire

## Abstract

**Background:**

Although the incidence, severity and mortality of *Clostridioides* (*Clostridium*) *difficile* infection (CDI) have been increasing, patients’ quality of life changes resulting from CDI have not been studied thoroughly. This study aimed at exploring the consequences of CDI on quality of life through patients’ perspective.

**Methods:**

An observational, cross-sectional study involving 350 participants with a self-reported CDI diagnosis was conducted through an online self-administered survey. Participants were grouped into those who had active disease (“Current CDI”) and those who had a history of CDI (“Past CDI”).

**Results:**

One hundred fifteen participants (33%) reported Current CDI and 235 (67%) reported Past CDI. A large majority of participants admitted that their daily activities were impacted by the infection (93.9% and 64.7% of Current and Past CDI respondents respectively, *p* < 0.05). Physical and psychological consequences of CDI were experienced by 63.5% and 66.1% of participants with active CDI. Despite the infection being cleared, these consequences were still frequently experienced in Past CDI cohort with similar rates (reported by 73.2% of respondents for both, physical consequences *p* = 0.08; psychological consequences *p* = 0.21). After the infection, 56.6% of respondents noted that post-CDI symptoms remained; 40.9% believed they would never get rid of them.

**Conclusions:**

While the societal burden of CDI is well described in the literature, our study is one of the first aimed at understanding the major burden of CDI on quality of life. Our results highlight the long-lasting nature of CDI and further reinforce the need for enhanced therapeutics in the prevention and treatment of this devastating infection.

## Background

*Clostridioides (Clostridium) difficile* (*C. difficile*) is a bacterium responsible for 15–25% of healthcare-acquired antibiotic associated diarrhea [1] and is one of the most common pathogens found in healthcare-associated infections in the United States (US) [2, 3].

The incidence, severity and mortality of *C. difficile* infection (CDI) have been increasing since 2000 in the US and the European Union with a steady rise over the last decade [4–7]. In 2011, there were an estimated 453,000 cases in the US resulting in approximately 83,000 recurrences and 29,300 deaths [8]. In 2013, the US Centers for Disease Control and Prevention designated CDI as an “urgent threat” requiring more monitoring and preventative actions to minimize its spread [9]. Moreover, in October 2016, the US Department of Health and Human Services set new goals to reduce the incidence of healthcare-associated infections. *C. difficile*’s goal was a 30% reduction prior to 2020 [10].

The clinical manifestations of CDI range from asymptomatic colonization, to mild or moderate diarrhea, but can also include fulminant colitis with toxic megacolon [11]. CDI can be devastating, with an estimated 30-day mortality ranging from 5.7% to 6.9% [12]. In addition, 20–30% of patients treated with either metronidazole or vancomycin experience one or more recurrences and following an initial recurrence, the risk of subsequent episodes increases to 40–60% [13]. Most studies of CDI focus on clinical outcomes including resolution of diarrhea, recurrence and mortality [13]; however, additional patient-centered outcomes need to be explored to optimally care for those suffering from this infection.

Several studies have already been published on CDI patient’s quality of life, using different methodologies. Among them, we can cite Madeo et al. [14] and Guillemin et al. [15] which were among the first studies to explore the perception and experience of CDI patients. They were performed on a limited number of patients (*n* = 15 and *n* = 24 respectively) and used a qualitative approach (interviews of patients with open-ended questions). In Heinrich et al. [16], authors used the SF-36v2 (Short Form 36-item Health Survey, version 2), a generic patient-reported outcome measure quantifying health-related quality of life. More recently, in Barbut et al. [17], the impact on patients was measured with the European Quality of life – 5 Dimensions – 3 Levels of severity (EQ-5D-3 L) a widely-used generic questionnaire. These studies describe a major impact of CDI on health-related quality of life for patients from the analysis of non-pathology-specific questionnaires. Despite a scarcity of quality of life data in those with CDI, chronic diarrhea in patients with HIV or those having received a kidney transplant has shown significant reductions in health-related quality of life, including decreased general well-being, social, and physical functioning [18, 19]. Only one study by Garey et al. [20] has attempted to develop and validate a questionnaire of quality of life taking into account the specificities of CDI. To date, the specific impact of CDI on patient-reported Health-Related Quality of Life (HRQoL) has not been thoroughly studied [19, 21, 22].

The purpose of this study is to explore the consequences of CDI on patient’s quality of life. We hypothesize that CDI will negatively impact patients physically, psychologically, socially and financially. Our primary goal is to qualitatively measure these factors and the resulting consequences during and after the infection to assess differences between CDI consequences at the time of infection and the long-term consequences of CDI. We aimed to evaluate the burden of CDI with the goal of helping physicians, healthcare providers and policy makers more accurately understand the needs for existing and novel therapies preventing and treating CDI.

## Methods

We conducted an observational cross-sectional study involving human subjects with self-reported CDI completing an online survey.

### Survey population

In order to recruit participants, the survey was advertised on CDI patients and survivors’ organizations websites, newsletters and Twitter accounts (C Diff Foundation [https://cdifffoundation.org/] and Peggy Lillis Foundation [http://peggyfoundation.org/]) as well as CDI-specific forums. Participants were also invited to participate at the Principal Investigators’ offices with a dedicated brochure that was handed to them.

Recruitment materials directed the participant to the link for the online survey. After providing informed consent to the study, all potential participants had to reply to 5 screening questions. Participants not residing in the US (Puerto Rico included), not having the legal adult age to consent (i.e. 19 in Alabama/Nebraska, 21 in Puerto Rico and 18 for any other state of the US) and participants without a self-reported current or past diagnosis of CDI were excluded. Participants were free to leave the survey at any time and did not receive any financial compensation for participation.

### Survey questions (see Electronic Supplementary Material A1)

Given the current shortage of literature considering the impact of CDI on patients’ quality of life, there is no formally accepted and validated HRQoL questionnaire specific to this disease state. In the past, Wilcox et al. used the EQ-5D-3 L questionnaire and the EQ visual analogue scale to explore HRQoL changes in CDI participants [23] and Garey et al. developed a health-related quality of life instrument (Cdiff32) based on the RAND Short-Form 36 (SF-36). Garey et al. demonstrated that there was no consensus towards assessment of CDI participants’ quality of life [20]. Therefore, our survey did not include these previously considered questionnaires. We designed our survey to *qualitatively* assess HRQoL as a way to guide future assessments whereas previous studies focused on *quantitative* assessment. Our questionnaire explores the consequences of CDI on the quality of life of participants, with questions adapted from existing questionnaires, participants’ experiences and experts’ opinions.

### Study design

Based upon the responses to the survey, participants were categorized into two groups: (i) those self-reportedly actively experiencing an episode of CDI when participating and (ii) participants reporting a history of CDI without a current episode (i.e. at least one episode of CDI in the past). Participants responded to demographic questions, questions about the CDI itself, including disease severity, treatments, symptoms and the number of recurrences. Additional questions examined HRQoL, work productivity, and consequences of CDI on participants quality of life. The survey was self-adaptive and the total number of items each participant answered varied based upon earlier replies. Most questions were categorical (e.g., yes/no) and were complemented with non-compulsory open-ended fields for comments.

### Instrument

The survey was conducted with LimeSurvey® (https://www.limesurvey.org/), a free and open source survey web application. Data collected during the survey were anonymous: the survey was designed to avoid collecting any identifiable information; also, the introductory consent form asked the participants not to provide any identifiable information. Data gathered were kept confidential. Every researcher involved in this study received human subject research’s education and signed a form attesting that they would not use the information they accessed to re-identify the participants.

### Data analysis

Descriptive statistics were used to summarize respondents’ answers. Analyses were performed on all participants and comparison were made between the following subgroups: (i) Participants with currently active CDI versus respondents with a CDI history and (ii) Past CDI participants having experienced at least one CDI recurrence versus Past CDI respondents having only experienced a primary episode. Qualitative answers were compared with χ2 test (or Fisher test depending on the size of each sub-group). All *p* values were considered exploratory. Data were analyzed using R version 3.4.1.

### Ethics, consent, and permissions

This survey was determined to be exempt from an Institutional Review Board (IRB) review under Category 2 (45 CFR 46.101(b)(2)) by Schulman IRB, an independent IRB, on August 3, 2017. Materials used by study participants such as recruitment materials, together with the questionnaire and participant consent were reviewed by Schulman IRB. Informed consent was obtained for participation in the study via an online informed consent form.

## Results

### Characteristics of surveyed population

**Fig. 1:**
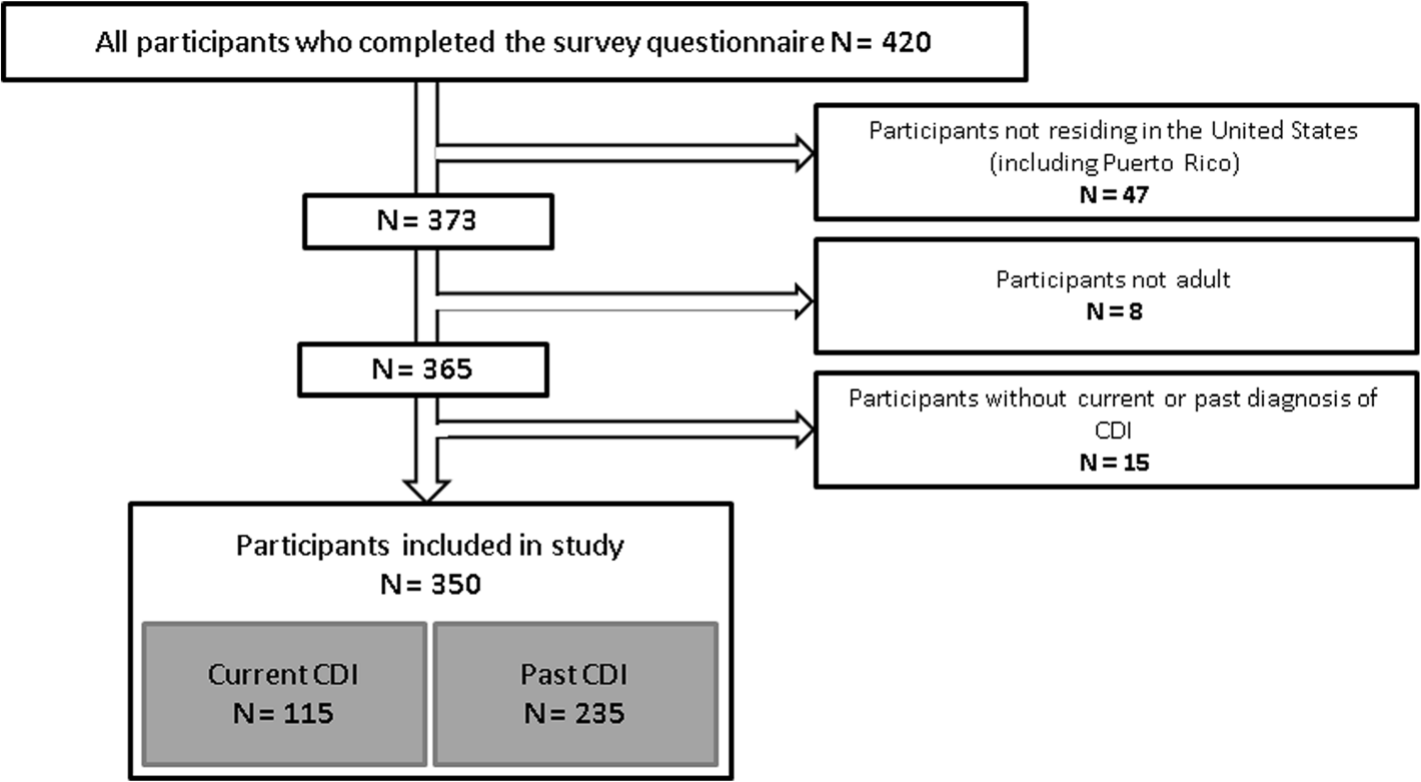
Construction of the study sample

The survey was completed by 420 individuals between August, 3rd, 2017 and November 17th, 2017. As 70 participants presented one or multiple exclusion criteria, the analysis was performed on 350 completed questionnaires. Of the 70 excluded participants, 47 were not residing in the US, 8 were under the legal age to participate and 15 did not report current or past diagnosis of CDI. Among those that met inclusion, 115 (32.9%) had active CDI at the time of the survey (“Current CDI” group) and 235 (67.1%) had CDI in the past (“Past CDI” group) (Fig. 1).

Participants’ demographic characteristics were similar in both cohorts. The majority of respondents were female (86.6%) and 55.7% were aged over 50. The two most common health insurances were private, covering approximately half of participants (50.6%), followed by Medicare (30.6%). Participants with no insurance represented a minority (3.4%) of participants (Table 1).

**Table 1:** Characteristics of survey participants (CDI: *Clostridioides difficile* infection)

	Total	Past CDI	Current CDI	*P*-value
Number of subjects	350 (100%)	235 (67.1%)	115 (32.9%)	
Age category, n (%)				
< 31	28 (8.0%)	15 (6.4%)	13 (11.3%)	0.2
31–40	56 (16.0%)	41 (17.4%)	15 (13.0%)	0.4
41–50	71 (20.3%)	45 (19.1%)	26 (22.6%)	0.5
51–60	78 (22.3%)	49 (20.9%)	29 (25.2%)	0.4
61–70	81 (23.1%)	58 (24.7%)	23 (20.0%)	0.4
71–80	22 (6.3%)	17 (7.2%)	5 (4.3%)	0.4
81–89	10 (2.9%)	7 (3.0%)	3 (2.6%)	1
> 89	4 (1.1%)	3 (1.3%)	1 (0.9%)	1
Gender n (%)				
Female	303 (86.6%)	204 (86.8%)	99 (86.1%)	1
Male	47 (13.4%)	31 (13.2%)	16 (13.9%)	1
Insurance coverage, n (%)				
Medicaid	30 (8.6%)	21 (8.9%)	9 (7.8%)	0.9
Medicare	107 (30.6%)	77 (32.8%)	30 (26.1%)	0.2
Veteran	9 (2.6%)	7 (3.0%)	2 (1.7%)	0.7
Private	177 (50.6%)	121 (51.5%)	56 (48.7%)	0.7
Other	66 (18.9%)	37 (15.7%)	29 (25.2%)	0.3
None	12 (3.4%)	4 (1.7%)	8 (7.0%)	0.02

### Characteristics of *C. difficile* infections experienced by participants

#### Current CDI group

Among respondents self-reportedly experiencing CDI at the time of the survey, the length of on-going CDI was variable: less than one third mentioned a CDI start date within the previous month (27.9%) while almost half (46.1%) indicated an infection lasting longer than 3 months. At the time of completing the survey, 84.3% of participants were receiving a treatment for CDI. Vancomycin (54.6%) or metronidazole (49.5%) were the most common treatments whereas fidaxomicin and fecal microbiota transplantation were seen less frequently in 17 (17.5%) and 10 (10.3%) respondents, respectively. Probiotics were commonly used (39.2% of respondents) (Table 2).

**Table 2:** Characteristics of participants’ *Clostridioides difficile* infection (CDI)

	Current CDI (*n* = 115)	Past CDI (*n* = 235)
Last CDI occurrence date (Past CDI) CDI occurrence start date (Current CDI)		
In the last week	8 (7%)	1 (0.4%)
Between 1 and 2 week(s) ago	7 (6.1%)	4 (1.7%)
Between 2 and 4 weeks ago	17 (14.8%)	2 (0.9%)
Between 1 and 3 months ago	30 (26.1%)	20 (1.7%)
Between 3 and 6 months ago	20 (17.4%)	24 (10.2%)
Between 6 and 12 months ago	16 (13.9%)	38 (16.2%)
More than 12 months ago	17 (14.8%)	146 (62.1%)
CDI treatments		
Subjects remembering their CDI treatment /currently treated for CDI	97 (84.3%)	229 (97.4%)
Metronidazole	48 (49.5%)	144 (62.9%)
Vancomycin	53 (54.6%)	187 (81.7%)
Fidaxomicin	17 (17.5%)	60 (26.2%)
Fecal microbiota transplant	10 (10.3%)	66 (28.8%)
Probiotics	38 (39.2%)	151 (65.9%)
Other treatment	4 (4.1%)	22 (9.6%)
Surgery		
GI surgery due to CDI	N/A	11 (4.7%)
Recurrences		
Subjects who have experienced at least one recurrence	N/A	105 (44.7%)

#### Past CDI group

**Fig. 2:**
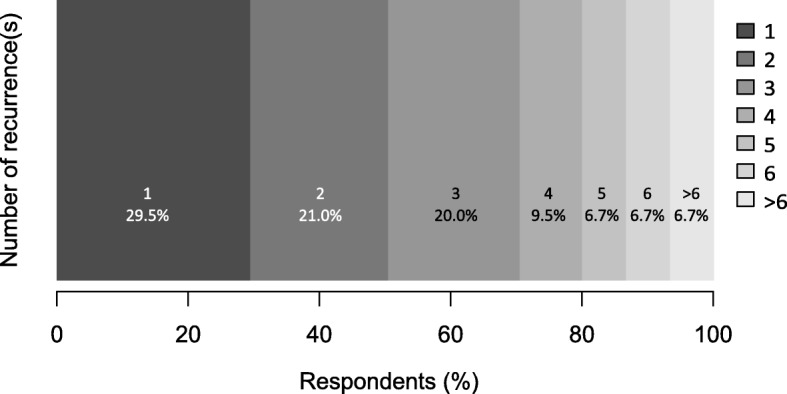
Number of recurrences reported by subjects with at least one recurrence in Past CDI group

The majority of Past CDI participants reported a significant amount of time since the prior infection with 62.1% estimating that their most recent episode took place greater than 12 months earlier and only 4.7% within the previous 3 months. Almost half (44.7%) of respondents reported at least one recurrence of the infection and 49.5% of those reporting at least 3 recurrences (Fig. 2).

A majority of participants (97.4%) remembered the antimicrobial therapies used to treat their CDI (Table 2). Trends in treatment seemed similar to those in the Current CDI cohort with the majority of Past CDI group receiving vancomycin (81.7%) and metronidazole (62.9%). Less were treated with fecal microbiota transplantation (28.8%) and fidaxomicin (26.2%). Likewise, probiotic usage was common with 65.9% of respondents having taken these supplements (Table 2).

### CDI impact on participants’ chronic conditions

Chronic medical co-morbidities were common in both cohorts with 44.3% and 53.2% of the Current CDI and Past CDI respondents respectively reported suffering from one or several chronic disease(s). The three most commonly reported conditions included depression (14.8% and 19.6% by Current and Past CDI respondents, respectively), high blood pressure (11.3% and 17.0%, respectively) and arthritis (11.3% and 17.4%, respectively). Irritable Bowel Syndrome (IBS) and Inflammatory Bowel Disease (IBD) were the most common chronic gastrointestinal diseases reported by participants (IBS cited by 8.7% and 20.9% and IBD reported by 9.6% and 11.1% of Current and Past respondents, respectively). When comparing Past with Current CDI, IBS was seen more frequently in the Past CDI cohort (*p* ≤ 0.05) (Table 3).

**Table 3:** Self-assessed impact of *Clostridioides difficile* infection (CDI) on participants’ chronic conditions

	Current CDI, n (%)	Negative impact of CDI, n (%)	Past CDI, n (%)	Negative impact of CDI, n (%)
	(*n* = 115)		(*n* = 235)	
≥ 1 chronic disease	51 (44.3%)	N/A	125 (53.2%)	N/A
Alzheimer’s disease or related disease	0 (0%)	N/A	0 (0%)	N/A
Arthritis	13 (11.3%)	9 (69.2%)	41 (17.4%)	24 (58.5%)
Cancer	4 (3.5%)	1 (25.0%)	6 (2.6%)	3 (50.0%)
COPD and allied conditions	5 (4.3%)	1 (20.0%)	12 (5.1%)	3 (25.0%)
Depression	17 (14.8%)	12 (70.6%)	46 (19.6%)	42 (91.3%)
Diabetes	7 (6.1%)	1 (14.3%)	12 (5.1%)	5 (41.7%)
Heart disease	3 (2.6%)	1 (33.3%)	11 (4.7%)	1 (9.1%)
High blood pressure	13 (11.3%)	5 (38.5%)	40 (17.0%)	15 (37.5%)
IBD	11 (9.6%)	9 (81.8%)	26 (11.1%)	23 (88.5%)
IBS	10 (8.7%)	8 (80.0%)	49 (20.9%)	43 (87.8%)
Osteoporosis	6 (5.2%)	2 (33.3%)	20 (8.5%)	8 (40.0%)
Stroke	2 (1.7%)	1 (50.0%)	3 (1.3%)	1 (33.3%)
Other	28 (24.3%)	N/A	58 (24.7%)	N/A

Participants were also asked whether they perceived that the CDI negatively impacted/impacts their chronic diseases. In both groups, IBS, IBD, arthritis and depression were considered by most participants to have worsened as a result of CDI. Depression seemed to be exacerbated very frequently in the Past CDI cohort with 42 of the 46 participants (91.3%) reporting that their CDI increased the activity and/or severity of their depressive symptoms (Table 3).

### Consequences of CDI

#### Physical and psychological consequences

Almost all Current CDI participants (93.9%) admitted that their daily activities were impacted by the infection. This alarming trend was seen in the Past CDI cohort, albeit less frequently (64.7%, *p* < 0.05). The impact of CDI was broad, affecting sleep patterns and respondents’ social life (cited by 73.9% and 79.1% of Current CDI respondents, respectively). Physical and psychological consequences of CDIs were experienced by approximately two thirds of participants with active CDI (63.5% and 66.1%, respectively). Despite the infection being cleared, these consequences were frequently experienced in the Past CDI cohort with similar rates of physical and psychological consequences compared with those with active CDI (reported by 73.2% of respondents for both, physical consequences *p* = 0.08; psychological consequences *p* = 0.21). After eradication of the infection (Past CDI group), remarkably, 56.6% of respondents noted that the post-CDI symptoms remained and 40.9% believed they would never get rid of them.

#### Impact of CDI on work activities

CDI has a major impact on patients’ professional lives. In the Current CDI group, 73.9% of participants identified an impact on their work activities with more than a third having to stop work as a result. Although the infections’ impact on professional life was significantly lower after clearance (46.0% of Past CDI respondents stated that their past infection continued to impact their work activities (*p* < 0.05)), almost half (47.2%) reported having stopped working while actively infected with 25.5% stopping work afterwards as a result of consequences from the episode. Once out of work, patients remained unable to perform their professional responsibilities for extended periods both during the infection (0–1825 days, mean: 118; median: 60) and after clearance (2–3000 days; mean: 310; median: 62.5).

#### Impact of the number of recurrences

**Fig. 3:**
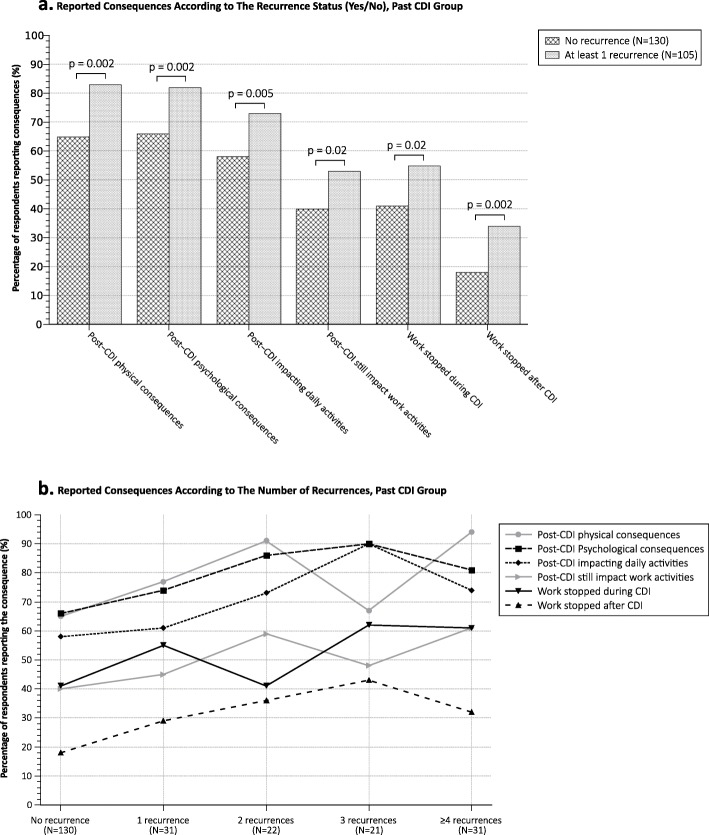
Impact of recurrences on CDI consequences, Past CDI group

When comparing those with any recurrence with patients having a single episode, those with recurrence had significantly higher rates of physical (*p* < 0.05) and psychological (*p* < 0.05) consequences, greater impacts on daily (*p* < 0.05) and work activities (*p* < 0.05) as well as more work interruptions (*p* < 0.05) (during and after the infection) (Fig. 3a).

When considering the number of reported recurrences, participants with a greater number of episodes showed a trend to reporting harmful consequences at higher rates (Fig. 3b).

#### Impact of CDI over time

**Fig. 4:**
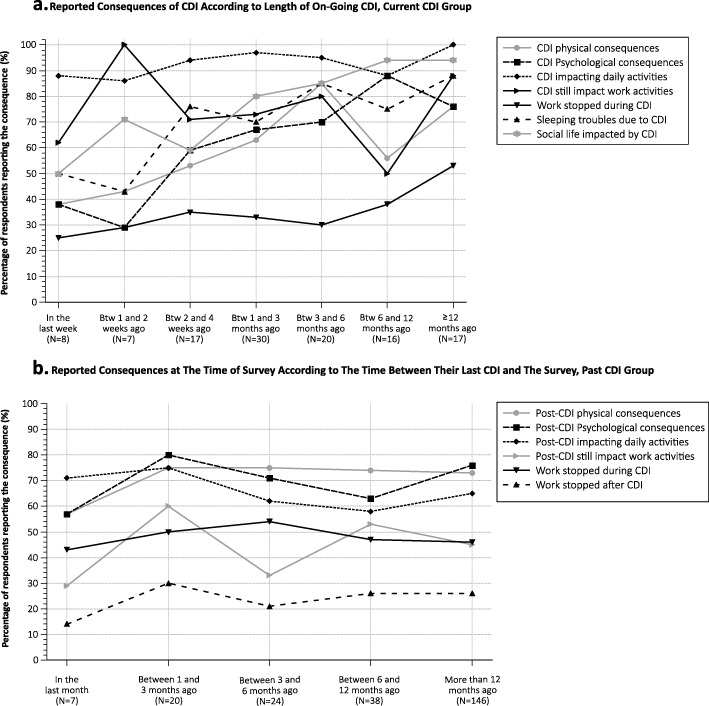
Impact of CDI over time

The consequences of CDI seemed influenced by the duration of infection with the Current CDI group reporting increased rates of physical, psychological, professional, social and impacts on daily activities with longer durations of disease (Fig. 4a). The impact of CDI on these factors also seems to last long after clearance of the infection. Indeed, the percentage of Past CDI participants reporting each of the evaluated consequences did not seem to wane with longer time periods since their last reported episode (Fig. 4b).

#### Financial impact of CDI

In both groups, approximately half of respondents were able to approximate how much out-of-pocket (OOP) money they spent caring for their disease (55.6% and 58.7% of Current and Past CDI respondents). On average, participants reported having spent $4355 and $8695 OOP in the Current CDI and Past CDI cohort, respectively (medians of $1000 and $2500 respectively) (See Electronic Supplementary Material A2).

### Fears associated to CDI

The psychological impact of CDI is high: 92.2% of Current CDI respondents identified being afraid that their CDI could get worse; 80% feared that certain foods might contribute to the worsening. 57.9% of those with CDI in the Past endorsed being frightened of a recurrence from certain foods. In both groups, any antibiotic intake was highly correlated to infection recurrence. Almost all respondents expressed fear of a future recurrence in the event they were to need a concomitant or future antibiotic treatment for other infectious disease. This fear was persistent after clearance of CDI with 97.0% of Past CDI respondents reporting this fear compared with 99.1% at the time of infection (*p* = 0.28). Overall, following infection, 87.2% of participants feared recurrences: this fear was not statistically stronger in patients with a history of recurrence compared to patients without any recurrence (89.5% vs 85.4%, *p* = 0.5). To the question “On a scale of 0 (least) to 10 (most), on a daily basis, how fearful are you that *C. difficile* would return?” 79.6% answered a score between 5 and 10 and 31.5% chose 10, the maximum.

## Discussion

The clinical impact of CDI on the digestive tract has been well documented [1, 24, 25]. As clinicians, it is important to gain insight into the patients’ perspectives on the disease state and its impact on not only their digestive tract, but also other elements of their lives. Our study shows the significant impact of both current CDI and a history of CDI on patients physical, psychological, social, professional and financial lives. Although participants with active disease seem to have the largest impact, this effect does not wane with time and patients are impacted well into the future.

Our results are consistent with previous studies evaluating the impact of CDI on patients’ quality of life. Here we propose a refined understanding of the patients’ perception presented in Madeo et al. and Guillemin et al. by collecting patient-reported outcomes from a larger cohort with both patients with active CDI or an history of CDI. We also offer a more precise view on key aspects to address in future HRQoL research to better take into account the specificities of CDI as some prior research was conducted with generic questionnaires. Similar to the studies published by Barbut et al. and Heinrich et al., our findings highlight a significant impact of the infection on active CDI patients’ lives with impairment of their daily activities and negative impact on their professional lives. We found more frequent consequences for participants who had experienced at least one recurrence. It had already been demonstrated in Barbut et al. that patients experiencing recurrences report significantly more mental consequence than first CDI patients. In our study, we observed a similar trend but physical, psychological and professional effects were also significantly more reported by participants with an history of CDI. These multifaceted sequelae impacting patients’ lives should be considered and addressed by practitioners. During a typical office visit, most of the time allotted is spent focused on eliciting the “classic” CDI symptoms such as changes in bowel habits, abdominal pains, fevers and fatigue. A short inquiry for psychological and personal sequelae might reveal issues that would otherwise be missed and this could validate that patients are not alone in their experience. Acknowledgement of these issues might also allow the patient the comfort of asking for help with these extra-intestinal symptoms and without these brief but impactful discussions many with CDI will likely continue holding these consequences within with negative future repercussions.

Our study is one of the first to assess the CDI consequences reported by participants after their infection and compare it with those actively infected. The long-lasting nature of this infection was highlighted by our study showing no difference between the Past and Current CDI cohorts when comparing the percent of those reporting physical or psychological sequelae. Some symptoms, such as IBS, were identified more frequently after CDI. This reconfirms the previously documented high frequency of IBS following CDI with an estimated 25% of patients having symptoms at least 6 months after completion of successful therapy [26]. The impact of IBS on patients with CDI can be profound with many worrying that their frequent loose stools are a recurrence or that their bodies are permanently changed as a result of this infection.

Fear and anxiety were reflected within our study by the high rate of respondents reporting that they were suffering from depression (nearly 3 times more than US adult population [27]) and the high frequencies reporting an exacerbation of those symptoms both during and after successful treatment of the infection. Multiple studies have highlighted the link between an altered microbiota and depression [28, 29] and it has already been demonstrated that depression is predictive of gastro-intestinal disorders [30, 31]. When considering CDI, one study from 2013 revealed that those with depressive disorders were at a 35% increased risk for disease development [32]. These results reinforce the importance of CDI prevention in another predisposed population. In addition, the anxiety associated with the sustainability of symptoms and the fear for recurrence might also play a role in the emergence and worsening of the depression. Indeed, the connection between the microbiota, depression and CDI is multifaceted and requires further assessment. Our findings should alter the clinical approach to CDI patients, focusing on both the pharmaceutical interventions but also patients’ anxiety and fears hopefully reducing the risk of new-onset or worsening depression.

Our results also revealed that Current and Past CDI had a negative impact on those with IBD. These findings are consistent with the previous literature documenting the increased risk of developing CDI in IBD patients, with more severe outcomes, including higher rates of hospitalization, colectomy and death [33–36]. IBD is known to impact patients physically, psychologically, professionally and financially [37–39]. Our study shows that CDI can have similar effects that were previously not fully appreciated. It is now the standard of care to test all patients with IBD presenting with a possible exacerbation for CDI given the increased incidence and severity in this population. By detecting this infection earlier and treating, this will hopefully minimize the down-stream effects that the combination of IBD and CDI might have.

Patients productivity was shown within our study to be significantly impacted by both Current and Past CDI. One might expect a high proportion of patients with active infection to have their professional lives impacted by the abdominal pains and diarrhea associated with CDI. However, identifying that 30.6% of patients with a history of CDI reported that this was still impacting their lives, long after the infection had been cleared, is very concerning. The significant economic burden of CDI on the US healthcare system has been previously well outlined [40–44]. However, the majority of studies evaluating the economic burden of the disease are limited to direct costs and do not include the decreased productivity of individuals in the work force. This is a concept that needs further evaluation to better understand the impact of this on individuals’ careers and their financial stability as our study identifies a major financial burden as well.

Our study is also the first to demonstrate CDI patients’ knowledge of the highly recurrent nature of infection and their awareness about the causal link between antibiotic use and the occurrence of CDI. In both cohorts, almost all participants expressed fear of a future recurrence in the event they were to need an antimicrobial treatment subsequent to their most recent episode of CDI. This shows a good understanding of the most common pathogenesis of this disease. Patients rely on medical practitioners to educate them about their disease, its etiology and management. Provider education benefits patients, and in the case of CDI, those patients should understand that following the infection, the use of antimicrobials should not necessarily be feared, but should come with caution and understanding that there must be a clear indication for antimicrobial usage, otherwise the risks for recurrent CDI outweigh the benefits.

The present study has several limitations. First, the questionnaire for the qualitative survey was not validated. However, it was adapted from the only previously published CDI-specific questionnaire and it was reviewed by experts in CDI for clinical applicability. Secondly, the patient replies were based on the perception of participants who classified themselves as having the infection at the time of the survey or in the past. This methodology might introduce a bias since the answers were not validated by healthcare professionals and may not represent the medical truth. We believe this to actually also be a strength of our study as our goal was to offer a deeper understanding of the *patients experience* with this infection, independent of the physician or healthcare practitioner’s perception. Thirdly, most questions offered only yes/no choices and not scales (e.g. Likert scale) which may lead to an overestimation of the rate of reported consequences as we certainly pooled together consequences with varying self-perceived severity. The analysis of the comments reported in the open-ended fields (some excerpts are shown in Table 4), although harder to interpret because of language subjectivity, merits further analysis to detect nuances in the reported outcomes. Future studies should use recommended methods for development and validation of Patient-Reported outcome assessments and health surveys [45, 46] to create a survey instrument that ensures it is appropriate for range of severity of CDI, and provides wording and response options that allow description of milder or less severe impacts of CDI. Finally, due to the data collection methodology, participants are not fully representative of the CDI population. Compared with data found in the literature, women and people under 50 seem over-represented in our sample which is probably due to the data being collected through an online web survey which lends to younger motivated patients who are technologically savvy. Also, our sample is much richer in patients with self-reportedly severe or multiply-recurrent forms of CDI (almost half of participants self-reported at least 2 CDI episodes). As a consequence, reported CDI treatments seem skewed with an increased usage of FMT and fidaxomicin, both of which are often used for those at high risk for recurrence or with a recurrence. This may be partially explained by the survey recruitment through two American Foundations who attract patients feeling they have severe forms of the infection or had multiple recurrences. This refractory population is also probably more motivated to complete a survey on CDI than those easily cured. Although this population is certainly more “severe” than a general population, we feel that the trends represented within our study are representative of broader concepts that apply to the general population with CDI, albeit possibly with a lower frequency.

**Table 4:** Illustrative quotes left by participants in the open-ended fields

Question about	Excerpt of comments left by participants
Psychological consequences	“I panic all the time and my anxiety is through the roof”
	“Never was depressed prior to infection. Now life as I knew it has drastically changed. Sometimes I feel I will never feel normal again.”
	“I have documented PTSD, anxiety and panic attacks directly related to the trauma of having a family wide endemic outbreak of C. diff effecting all members of my family”
	“I have felt suicidal because of it many times.”
Physical consequences	“I don’t have the energy anymore to be physical, I am too tired to do anything!”
	“My sleep cycle is messed up, due to always being in so much pain. I have trouble falling and staying asleep, then during the day I often feel tired.”
	“Lost 50 pounds all muscle tone lost skin and bones malnutrition”
	“I throw up for no reason (even if I havent eaten yet)”
Relational impact	“I feel like everyone is afraid of me.”
	“My family treats me like a leper.”
	“Other people don’t seem to understand how my life changed post CDif.”
	“There is a social stigma associated with c-diff. People never consider you “cured”. I am sometimes excluded from activities due to the type of illness it is.”
Adaptation impact	“I have to wear adult diapers. If I don’t wear them, then I have it on my mind through the night that I will have another accident.”
	“I can’t eat anything other than the BRAT diet!”
	“I don’t travel far from home anymore”
	“Work going to outdoor summer activities like parks, hiking, amusement parks, biking, swimming. Anything that takes me away from a bathroom or a place to clean up and change clothes after accidents”
Professional and financial impact	“was already in financial trouble. I am on the verge of loosing everything.”
	“I lost my job of 12 years mostly because my productivity went down”
	“I can’t work at all”
	“After 6 months of being on disability I am bankrupt. It has ripped through our house financially and caused “

## Conclusions

While the societal burden of CDI is well described in the literature, our study is one of the first aimed at understanding the major burden of CDI on patients’ quality of life. Our results demonstrate a significant burden on patients’ quality of life caused by *C. difficile* at the time of the infection and afterwards. These results clearly define the need for development of new strategies for better prevention, treatment, communication and education from providers both at the time of active CDI and in the future.

## Supplementary information

**Additional file 1.** Survey Questionnaire.

**Additional file 2.** Financial impact of *Clostridioides difficile* infection (CDI), reported CDI out-of-pocket expenses (OOP), Current CDI (*N* = 64) And Past CDI (*N* = 138), US Dollar – Boxplot Representation.

## Data Availability

The data that support the findings of this study are not publicly available.

## Abbreviations

*C. difficile*: *Clostridioides difficile*
CDI: *Clostridioides difficile* infection
EQ-5D-3 L: European Quality of life – 5 Dimensions – 3 Levels of severity
HIV: Human Immunodeficiency Virus
HRQoL: Health-related quality of life
IBD: Inflammatory Bowel Disease
IBS: Irritable Bowel Syndrome
IRB: Institutional Review Board
OOP: Out-of-pocket
SF-36: Short Form 36-item Health Survey
SF-36v2: Short Form 36-item Health Survey, version 2
US: United States
