# Supplementary material for: Perception of quality of life in people experiencing or having experienced a Clostridioides difficile infection: a US population survey
Source: J Patient Rep Outcomes. 2020 Feb 19;4:14. doi: 10.1186/s41687-020-0179-1 (PMC7031450; doi:10.1186/s41687-020-0179-1)
Supplement: Supplementary file 1 — Additional file 1. Survey Questionnaire. [file 41687_2020_179_MOESM1_ESM.docx]

## **SOM1. Survey Questionnaire**

### Screening questions

1. Where do you live in the USA? (*compulsory*)
   1. Alabama or Nebraska
   2. Puerto Rico
   3. Any other State of the USA
   4. None of the above *(end of the questionnaire)*
2. How old are you? *(compulsory)*

*List of choices*:

- 1. According to the participants first answer *(if they answer so, they will be excluded):*
     1. <19 (for participants residing in **Alabama** or **Nebraska**)
     2. <21 (for participants residing in **Puerto Rico**)
     3. <18 (for participants residing in any other State of the USA)
  2. According to the participants first answer:
     1. 19-30 (for participants residing in **Alabama** or **Nebraska**)
     2. 21-30 (for participants residing in **Puerto Rico**)
     3. 18-30 (for participants residing in any other State of the USA)
  3. 31-40;
  4. 41-50;
  5. 51-50;
  6. 51-60;
  7. 61-70;
  8. 71-80;
  9. 81-89;
  10. >89

1. What is your sex? *(compulsory)*
   1. M/F
2. Which insurance coverage do you have? *(multiple choices possible, not compulsory)*
   1. Medicaid
   2. Medicare
   3. Veteran
   4. Private
   5. Other
   6. none
3. Are you in one the following situations?
   1. I experienced a *Clostridium difficile* infection in the past (go to CDI history questionnaire)
   2. I am currently experiencing a *Clostridium difficile* infection (go to current CDI questionnaire)
   3. I am not in the two above situations (end of the questionnaire)

### CDI history questionnaire

1. When did your last *Clostridium difficile* infection occur? *(compulsory, add the following note: we are considering the initial episode, not the potential relapses)*
   1. More than 12 months ago
   2. Between 6 and 12 months ago
   3. Between 3 and 6 months ago
   4. Between 1 and 3 months ago
   5. Between 2 and 4 weeks ago
   6. Between 1 and 2 weeks ago
   7. In the last week
2. Do you remember which treatment you received for your *Clostridium difficile* infection? *(compulsory)*
   1. YES/NO
   2. (*if YES)*: What was/were your treatment(s) for your *Clostridium difficile* infection? *(compulsory)*
      - Metronidazole
      - Vancomycin
      - Fidaxomicin
      - Fecal transplant
      - Probiotics
      - Other (*free text if selected, not compulsory)*
      - I do not know or do not want to reply
3. Did you experience recurrences after your last *Clostridium difficile* infection? *(compulsory)*
   1. YES/NO

Information: Recurrence is present when CDI re-occurs within 8 weeks after the onset of a previous episode, provided the symptoms from the previous episode resolved after completion of initial treatment. (*Debast et al., 2014; Surawicz et al., 2013*)

- 1. If yes, how many recurrences did you experience since your last initial episode? *(number field)*
  2. If yes, when was your last recurrence?
     1. More than 12 months ago
     2. Between 6 and 12 months ago
     3. Between 3 and 6 months ago
     4. Between 1 and 3 months ago
     5. Between 2 and 4 weeks ago
     6. Between 1 and 2 weeks ago
     7. In the last week

1. Did you undergo gastro-intestinal surgery because of your *Clostridium difficile* infection? *(compulsory)*
   1. YES/NO
2. Do you have any chronic disease(s)? *(compulsory)*
   1. YES/NO
   2. If yes, what chronic disease(s) do you have? (compulsory*)*
      1. IBS
      2. IBD
      3. High blood pressure
      4. Alzheimer's disease or other dementias
      5. Heart disease
      6. Depression
      7. Arthritis
      8. Osteoporosis
      9. Diabetes
      10. COPD and allied conditions
      11. Cancer
      12. Stroke
      13. Other: (*free text if selected, not compulsory)*
   3. For each chronic disease ticked: Would you say that *Clostridium difficile* worsened your [chronic disease]? (If yes, free text, not compulsory), please list the consequences of *Clostridium difficile* on your chronic disease
3. Do you have post-CDI physical consequences that you attribute to your last *Clostridium difficile* infection? *(compulsory)*
   1. YES/NO
   2. If yes, please explain (*free text only if yes, not compulsory)*
4. Do you have psychological consequences of your last *Clostridium difficile* infection? *(compulsory)*
   1. YES/NO
   2. If yes, please explain *(free text only if yes, not compulsory)*
5. Does this past *Clostridium difficile* infection still affect your daily activities? *(compulsory)*
   1. YES/NO
   2. If yes, please explain (*free text only if yes, not compulsory)*
6. Does your last *Clostridium difficile* infection still affect your health in any other way? *(compulsory)*
   1. YES/NO
   2. If yes, please list all other consequences (*free text only if yes, not compulsory)*
7. Do you think that you got rid of your post-CDI symptoms? *(compulsory)*
   1. YES/NO
   2. If no, please explain why (*free text only if no, not compulsory)*
   3. If no, do you think that you will get rid of your post-CDI symptoms?
      1. YES/NO
      2. If no, please explain why (*free text only if no, not compulsory)*
8. Are you afraid that your last *Clostridium difficile* infection could come back again? *(compulsory, only if initial infection or recurrence within the last 3 months)*
   1. YES/NO
9. Are you afraid that the next time you will need antibiotics, your *Clostridium difficile* infection will appear again? *(compulsory)*
   1. YES/NO
10. Are you afraid that certain foods will trigger again the symptoms of *Clostridium difficile* infection? *(compulsory)*
    1. YES/NO
11. Do you know how much out-of-pocket money you spent on medical care for your *Clostridium difficile* infection? *(compulsory)*
    1. YES/NO
    2. If yes, how much? *(free text only if yes, not compulsory)*
12. Does your post-CDI symptom(s) still affect your work activities?
    1. YES/NO
    2. If yes, please explain (*free text only if yes, not compulsory)*
13. Have you stopped working because of your last *Clostridium difficile* infection?
    1. Yes, during the time of the infection
       1. If yes, how many days have you stopped working? *(number field, not compulsory)*
    2. Yes, after the infection, because of the post-CDI symptoms
       1. If yes, how many days have you stopped working? *(number field, not compulsory)*
    3. No
14. On a scale of 0 (least) to 10 (most), on a daily basis, how fearful are you that *Clostridium difficile* would return? (compulsory)
    1. selection on a scale of 1 to 10
15. Are there any other consequences from your last *Clostridium difficile* infection that affects your life today?
    1. YES/NO (compulsory)
    2. If yes, please explain *(free text only if yes, not compulsory)*

### Current CDI questionnaire

1. When did your *Clostridium difficile* infection start? *(compulsory)*
   1. More than 12 months ago
   2. Between 6 and 12 months ago
   3. Between 3 and 6 months ago
   4. Between 1 and 3 months ago
   5. Between 2 and 4 weeks ago
   6. Between 1 and 2 weeks ago
   7. In the last week
2. Are you currently treated for your *Clostridium difficile* infection? *(compulsory)*
   1. YES/NO
   2. (*if YES)*: What is/are your current treatment(s) for your *Clostridium difficile* infection? *(compulsory)*
      - Metronidazole
      - Vancomycin
      - Fidaxomicin
      - Fecal transplant
      - Probiotics
      - Other (*free text if selected, not compulsory)*
      - I do not know or do not want to reply
3. Do you have any chronic disease(s)? *(compulsory)*
   1. YES/NO
   2. If yes, what chronic disease(s) do you have? (compulsory*)*
      1. IBS
      2. IBD
      3. High blood pressure
      4. Alzheimer's disease or other dementias
      5. Heart disease
      6. Depression
      7. Arthritis
      8. Osteoporosis
      9. Diabetes
      10. COPD and allied conditions
      11. Cancer
      12. Stroke
      13. Other: (*free text if selected, not compulsory)*
   3. For each chronic disease ticked: Would you say that *Clostridium difficile* worsened your [chronic disease]? (If yes, free text, not compulsory) please list the consequences of *Clostridium difficile* on your chronic disease
4. Do you have physical consequences that you attribute to your *Clostridium difficile* infection? *(compulsory)*
   1. YES/NO
   2. If Yes, please explain *(free text, not compulsory)*
5. Do you have psychological consequences that you attribute to your *Clostridium difficile* infection? *(compulsory)*
   1. YES/NO
   2. If Yes, please explain *(free text, not compulsory)*
6. Does your *Clostridium difficile* infection affect your daily activities? *(compulsory)*
   1. YES/NO
   2. If yes, please explain *(free text only if yes, not compulsory)*
7. Does your *Clostridium difficile* infection affect your health in any other way? *(compulsory)*
   1. YES/NO
   2. If yes, please list all other consequences *(free text only if yes, not compulsory)*
8. Are you afraid that your *Clostridium difficile* infection could get worse in the future? *(compulsory)*
   1. YES/NO
9. Are you afraid that the next time you’ll need antibiotics, your *Clostridium difficile* infection would appear again? *(compulsory)*
   1. YES/NO
10. Are you afraid that certain foods would worsen your *Clostridium difficile* infection? *(compulsory)*
    1. YES/NO
11. Because of your *Clostridium difficile* infection, have you had trouble sleeping? *(compulsory)*
    1. YES/NO
    2. If yes, please explain *(free text only if yes, not compulsory)*
12. Because of your *Clostridium difficile* infection, have you experienced challenges with social interactions or fear of socialization? *(compulsory)*
    1. YES/NO
    2. If yes, please explain *(free text only if yes, not compulsory)*
13. Do you know how much out-of-pocket money you spent on medical care for the *Clostridium difficile* infection? *(compulsory)*
    1. YES/NO
    2. If yes, how much? *(free text only if yes, not compulsory)*
14. Does your *Clostridium difficile* infection affect your work activities? *(compulsory)*
    1. YES/NO
    2. If yes, please explain *(free text only if yes, not compulsory)*
15. Have you stopped working because of your *Clostridium difficile* infection? *(compulsory)*
    1. YES/NO
16. Are there any other consequences from your *Clostridium difficile* infection that affect your life? *(compulsory)*
    1. YES/NO
    2. If yes, please explain *(free text only if yes, not compulsory)*
