# Supplementary figures and images for: Perception of quality of life in people experiencing or having experienced a Clostridioides difficile infection: a US population survey
Source: J Patient Rep Outcomes. 2020 Feb 19;4:14. doi: 10.1186/s41687-020-0179-1 (PMC7031450; doi:10.1186/s41687-020-0179-1)

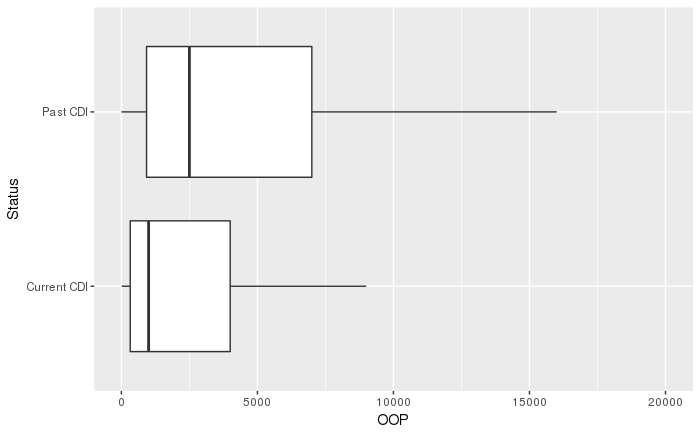

Supplement: Supplementary file 2 — Additional file 2. Financial impact of Clostridioides difficile infection (CDI), reported CDI out-of-pocket expenses (OOP), Current CDI (N = 64) And Past CDI (N = 138), US Dollar – Boxplot Representation. [file 41687_2020_179_MOESM2_ESM.tif]
